# 160. Urgent Care Prescriber Perspectives on Antibiotic Prescribing During the COVID-19 Pandemic

**DOI:** 10.1093/ofid/ofab466.362

**Published:** 2021-12-04

**Authors:** Brooke Betts, David R Ha, Marisa Holubar, Marisa Holubar, Maja Artandi, Sharon Onguti, Ian Nelligan

**Affiliations:** 1 Stanford Health Care, Stanford, California; 2 Stanford Antimicrobial Safety and Sustainability Program, Stanford, California; 3 Stanford University School of Medicine, Stanford, CA; 4 Stanford University, Palo Alto, California

## Abstract

**Background:**

Urgent care practices were significantly impacted by the COVID-19 pandemic. Studies conducted early in the pandemic demonstrated dramatic decreases in outpatient antibiotic prescribing, particularly amongst agents typically used for respiratory infections. We observed a 33% decline in urgent care antibiotics prescribing during the COVID-19 pandemic in our urgent care clinics. We investigated the prescriber experience to elucidate factors influencing antibiotic use for respiratory conditions during the COVID-19 pandemic at two academic urgent care clinics.

**Methods:**

We employed a mix method approach, first distributing a survey to all full-time prescribers. We then followed up with qualitative interviews (12 of 22 prescribers) which was conducted by a single, trained interviewer using a standardized guide. Interviews were recorded and transcribed verbatim. Each transcription was independently reviewed and coded by two blinded investigators using standardized themes and adjudicated by a third investigator for stability, robustness, and interrater reliability. Individually, researchers identified and coded key themes and statements. These themes were then discussed as a group and combined where they shared meaning. This project was reviewed and deemed to be non-human subjects research by the Stanford University School of Medicine Panel on Human Subjects in Medical Research.

**Results:**

A total of 20 of the 22 prescribers (13 MDs and 9 APPs) completed the survey (91% response rate). Notably, only 25% of prescribers agreed that COVID-19 had changed their antibiotic prescribing practices for patients with respiratory infections despite objective data that all prescribed less. In the qualitative interviews, we identified four major themes impacting the appropriateness of antibiotic prescribing practices as shown in Table 1.

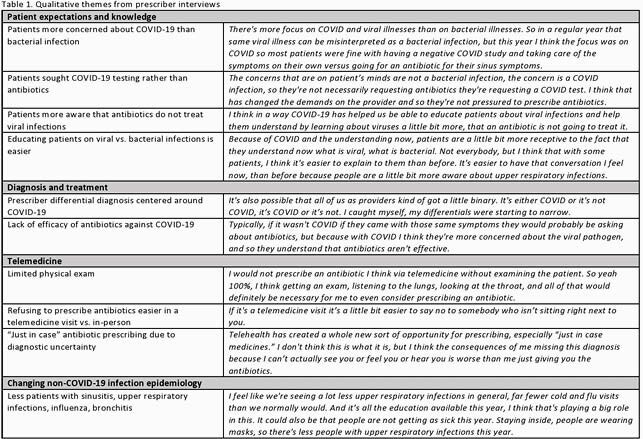

**Conclusion:**

Urgent care prescribers attributed a decrease in antibiotic prescribing during COVID-19 to changes in patient expectations and knowledge base, a switch to telemedicine-based encounters, and changing epidemiology. These shifts could be utilized by outpatient antimicrobial stewardship efforts to sustain low prescribing rates for conditions in which antibiotics are generally not indicated.

**Disclosures:**

**Marisa Holubar, MD, MS**, Nothing to disclose

